# The role of leisure-time physical activity in youth for lifelong activity—a latent profile analysis with retrospective life course data

**DOI:** 10.1007/s12662-023-00884-9

**Published:** 2023-05-26

**Authors:** Lars Lenze, Claudia Klostermann, Julia Schmid, Markus Lamprecht, Siegfried Nagel

**Affiliations:** 1 Institute of Sport Science, University of Bern, Bremgartenstraße 145, 3012 Bern, Switzerland; 2 University of Teacher Education, University of Applied Sciences and Arts Northwestern Switzerland, 5210 Windisch, Switzerland; 3 Lamprecht und Stamm Sozialforschung und Beratung, 8032 Zurich, Switzerland

**Keywords:** Sport participation, Youth development, Person-oriented approach, Pattern, Time-related and context-related aspects

## Abstract

Considering the positive health effects of leisure-time physical activity (LTPA), youth is an important life stage to promote lifelong LTPA. However, the stability of LTPA over the life course is low, and specific predictors of LTPA in youth for lifelong activity have some shortcomings, e.g. neglecting the interacting factors of LTPA within individuals. Therefore, from a person-oriented approach, patterns of LTPA behaviour in youth considering time- and context-related aspects and their relationships with lifelong LTPA were investigated. Life course data from *n* = 1519 Swiss inhabitants aged between 25 and 76 years were recorded retrospectively using a validated questionnaire (CATI method). *Latent profile analyses* were used to find the optimal profile solution and for the association with lifelong LTPA *auxiliary conditional effect models* (controlled for age) were applied. Six distinct patterns emerged. Overall, mostly inactive youth are also the least active in adulthood, whereas several other patterns are associated with a mainly continuous LTPA throughout adulthood. More precisely, multiple constellations in youth occurred to be physically active in at least 80% of the years in adulthood: (1) early starters regarding LTPA in a rather self-organised setting but not with many different LTPAs; (2) late entrants with a variety of different activities and organisational settings; or (3) a high expression in every variable investigated. Consequently, there is not just one type of LTPA behaviour in youth linked to lifelong activity, which indicates that certain aspects of LTPA in youth can be compensated by each other. Implications for LTPA promotion can be derived.

**Supplementary Information:**

The online version of this article (10.1007/s12662-023-00884-9) contains supplementary material, which is available to authorized users.

## Introduction

Promoting regular and long-lasting leisure-time physical activity (LTPA) for a wide range of the population is a goal among various societal actors given the long-term health-promoting effects (Reiner, Niermann, Jekauc, & Woll, 2013) and the high health costs caused by physical inactivity (Ding et al., 2016). For this purpose, youth is a decisive life stage to shape later health behaviour (Sawyer et al., 2012) including lifelong LTPA (Kirk, 2005), which is supported by the general life course approach (Bernardi, Huinink, & Settersten, 2019). Time-related interdependencies over the life course are assumed, which means that not only the recent past influences the present but also the far-away past (see *path dependency *in Bernardi et al., 2019). Hence, no single time points or single selected explanation factors can be examined; rather, their various interdependencies must be examined. Thus, it is important to consider a long period in order to understand and explain later outcomes in life. However, in studies investigating the association between differentiated factors of LTPA in youth and later LTPA in life, the measurement of lifelong LTPA was mostly recorded for only one time point in (young) adulthood. Thus, there is no information on long-term or even lifelong LTPA throughout adulthood. Another characteristic of the life course approach is the amount of time spent in certain situations, which is highly relevant for the further course of life (Mayer, 1990) and for LTPA: the longer continuously active in life, the lower the chance of becoming inactive (Engel and Nagel, 2011). Persistent exercising for at least three years in youth is a predictor for being later active in life (Batista et al., 2019). In addition, a long physically active period in youth can also be achieved by early entry into LTPA, which is also predictive for later LTPA in life (Kjønniksen, Anderssen, & Wold, 2009). The aforementioned time-related high involvement in LTPA, also referred to as the depth of activities, can be further supported by a high weekly frequency of LTPA in youth, which seems relevant for later LTPA in life as well (Batista et al., 2019).

In addition to time-related aspects, from a human development perspective, the context plays a decisive role to understand and explain behaviour and development (e.g. Lerner, 2006). This is also shown for LTPA in youth: specific types of activities (e.g. endurance sports, Kjønniksen, Anderssen, & Wold, 2008; Tammelin, Näyhä, Hills, & Järvelin, 2003), organised and often club-based activities (Kjønniksen et al., 2009; Scheerder et al., 2006), self- and non-organised activities (Cleland, Dwyer, & Venn, 2012; Scheerder et al., 2006) and competitions (Batista et al., 2019) are related to LTPA later in life. The number of different contexts represented by the number of different activities in youth, also called breadth of activities, is also promising for LTPA later in life (Engström, 2008; Cleland et al., 2012; Kjønniksen et al., 2008). Consequently, individuals choose environments to participate in LTPAs in specific contexts, which leads to multiple combinations of activities and the exposure to various contexts (Agans and Geldhof, 2012). Thus, there is not only one LTPA in one context in youth for conducive development (cf. Coakley, 2011); rather, activities and contexts are interrelated, and thus interactions within individuals occur (e.g. football can be played in a club or self-organised; cf. Gut, Schmid, & Conzelmann, 2020, Zarrett et al., 2009). There are various such constellations in a population, i.e. interindividual differences (e.g. some play football in a club, others play self-organised and others do both; e.g. Zarrett et al., 2009). Consequently, analysing separate variables or predictors of LTPA in youth fall short, also due to including only aggregate values on group levels (cf. Zarrett et al., 2009) and thus neglecting possible compensation effects (e.g. high values in certain variables can counterbalance low levels in other variables for lifelong LTPA) and synergistic effects (e.g. high levels of variables strengthen each other for lifelong LTPA) within an individual. Therefore, the person-oriented approach describing patterns of LTPA in youth seems promising (Bergman, Magnusson, & El-Khouri, 2003). Empirical studies that describe such patterns of LTPA in youth differ regarding the variables used for person-oriented analyses: (1) specific types or categories of activities (Agans and Geldhof, 2012; Borgers et al., 2015); (2) organisational settings differentiating between organised and self-organised settings (Gut et al., 2020; Gut, Schmid, Imbach, & Conzelmann, 2022; Lawler, Heary, & Nixon, 2017); and (3) the breadth (number of different activities) and depth (frequency/duration of activities) of activities (Agans, Johnson, & Lerner, 2017). Klostermann and Nagel (2011) included the breadth and depth as well as the organisational setting but for the first 39 years of life—not in youth. However, the reported studies investigated patterns of LTPA in youth (except Klostermann and Nagel, 2011) but not the relationship with lifelong LTPA.

Several attempts at explanation were made to explain the relationship between LTPA in youth and lifelong LTPA. Telama (2009) formulated therefore hypotheses. For example, the *carry-over value hypothesis* means that specific types of physical activities learned in early years are continued in adulthood, also called lifestyle activities. In addition, the *ability and readiness hypothesis* indicates that early experiences of activities with basic skills facilitate the continuation of being physically active or to re-enter in the same or other activities. This hypothesis from Telama is similar to the early sampling approach (Côté, Baker, & Abernethy, 2007), not for achieving later success in one activity but for later recreational participation, or in other words, for lifelong activity. Therefore, experiences with a broad range of activities (‘sampling’) in youth helps during the life course to start new activities, to continue given activities or to re-enter already practiced activities.

In summary, when investigating the relationship between LTPA in youth and lifelong LTPA, the following research desiderata should be considered: (1) no single time points or single selected explanation factors but rather the entire life course and interdependencies between time and explanation factors, (2) time-related and context-related information to represent LTPA behaviour in youth and (3) person-oriented analysis methods allowing for intra- and interindividual interactions and differences. Moreover, (4) sociodemographic aspects such as sex, educational level and age are well-known differentiation aspects of LTPA in youth (e.g. for Switzerland: Lamprecht, Bürgi, & Stamm, 2020) and should thus be taken into account. In this study, the aforementioned research desiderata are considered and three research questions (RQ) are defined:

### RQ 1:

Which patterns of time-related and context-related information about LTPA emerge in youth?

### RQ 2:

How can the emerged patterns further be described in regard to sociodemographic variables and specific types of LTPAs?

### RQ 3:

To what extent are the patterns from youth associated with lifelong LTPA?

## Materials and methods

### Design and sample

This study is part of a project funded by the Swiss National Science Foundation and in collaboration with the federal survey “Sport Schweiz 2020”. LTPA over the whole life course was recorded with a retrospective and validated questionnaire. More precisely, a retrospective telephone survey with computer-assisted telephone interviews (CATI method) of Swiss inhabitants aged between 25 and 76 years was conducted in 2019. The random sample was recruited via the Federal Statistical Office and with persons from the panel of the survey institute. The questionnaire used is a further development of previous studies investigating LTPA during the life course (e.g. Klostermann and Nagel, 2011) and was tested qualitatively and quantitatively in multiple waves, including a separate reliability check with the test–retest method (*n* = 29; for a detailed description for the validation of the questionnaire, see Lenze, Klostermann, Lamprecht, & Nagel, 2021). Using Krippendorff’s *α* to consider multiple scale levels for the reliability check, all variables used showed good values (*α* > 0.80; Krippendorff, 2018; see specific values in Supplementary Table 1).

After conducting the survey, the data of each participant were checked carefully for internal consistency and discrepancies, which led to the exclusion of 222 participants, resulting in a final sample of *n* = 1519 (*n* = 569 from the Federal Statistical Office; *n* = 950 from the panel of the survey institute).

The study was conducted according to the guidelines of the Declaration of Helsinki and was approved by the Ethics Committee of the University of the University of Teacher Education, University of Applied Sciences and Arts Northwestern Switzerland (30 January 2019). Informed consent was obtained from all subjects involved in the study.

### Measures

LTPA is understood as physical activities including exercise, sport and unstructured recreation and excludes domestic, occupational, and commuting physical activity (Khan et al., 2012; see Lenze et al., 2021 for a detailed description). In this article, the term *youth* relates to the first 20 years of one’s life. To build patterns of LTPA behaviour in youth (RQ 1), four indicators considering time-related and context-related information were included and recorded up to the age of 20:

#### Number of regularly active years.

The depth of activities was measured by regularly active years in terms of LTPA between 3 and 20 years of age. The term ‘regular’ refers to at least once a week. Thus, each regularly active year in the aforementioned age range was added per participant.

#### Number of different activities practiced.

To cover the breadth of activities, each LTPA practiced at least multiple times per year for more than one year was included. The maximum number of different LTPAs was set to five.

#### *Self-organised activities*.

LTPAs practiced regularly by oneself or with friends reflect one part of the organisational setting. This indicator was dummy coded if this organisational form was practiced (1) or not (0) in youth. The term ‘self-organised’ is equivalent to ‘informal’ or ‘non-organised’.

#### Organised activities.

Regular club-based LTPAs or at private sports providers (e.g. fitness centre, yoga or dance studio) comprise this part of the organisational setting. This was coded as a dummy variable if activities were practiced organised (1) or not (0) in youth.

To enrich the patterns with further relevant information regarding aspects affecting LTPA behaviour (RQ 2), sociodemographic variables and categories of specific types of LTPAs were included. *Sex, age at the time of the survey *and *educational level* were considered. The educational level is represented by a 5-level variable (1 compulsory school; 2 secondary school/lower professional education; 3 higher professional education leaving certificate; 4 technical college; 5 university). To provide insights into which types of LTPA were practiced in the respective pattern, ten *categories of types of LTPAs* from Sudeck, Lehnert, & Conzelmann (2011) were used (1 walking and endurance activities; 2 fitness; 3 gymnastics and multisport activities; 4 athletics; 5 compositional–creative activities; 6 release-oriented activities; 7 outdoor- and mountain activities; 8 sports games; 9 martial arts; 10 equestrian).

Concerning lifelong LTPA (RQ 3), an *index of lifelong LTPA* with the ratio of physically active and inactive years in adulthood was developed for each participant. Due to the broad age range at the time of the survey, a comparable score was calculated from 21 years until the age at time of the survey of each person (ratio of regularly active years divided by the years of life from 21 upwards). This index reflects LTPA throughout adulthood.

### Data preparation and analyses

To identify patterns of LTPA in youth (RQ 1), latent profile analyses (LPA) with four indicators were conducted (Masyn, 2013). This procedure allows to integrate continuous and categorical variables as indicators. In this study, two indicators (*number of regularly active years *and* number of different activities practiced*) were used as continuous variables, whereas the other two indicators (*self-organised activities *and* organised activities*) were applied as categorical variables. One to eight profiles were calculated. To find the optimal number of profiles, a mix between content-related and statistical criteria were used. The content-related criteria included theory-based considerations, clear qualitative differences between profiles, the principle of parsimony and a lack of small profiles (< 5% of the sample) (Masyn, 2013; Morin and Wang, 2016). In contrast, the statistical criteria comprised the screening of the log likelihood value (LL), Akaike information criterion (AIC), consistent AIC (CAIC), Bayesian information criterion (BIC), sample-sized adjusted BIC (SABIC), bootstrap likelihood ratio test (BLRT) and the Vuong–Lo–Mendell–Rubin likelihood ratio test (VLMR) (Marsh, Lüdtke, Trautwein, & Morin, 2009; Masyn, 2013; Morin and Wang, 2016). Due to large sample sizes, adding a profile often yields significant BLRT and VLMRT as well as lower AIC, CAIC, BIC, and SABIC values (Marsh et al., 2009). Thus, the separate fit-indices provide no adequate measures and the elbow plot of the fit-indices must be taken into account (Morin & Wang, 2016). For the further description of the patterns (RQ 2), the means of the sociodemographic variables (sex, age, educational level) per pattern were taken and the Wald test was used to identify significant differences between the patterns (*p*-value < 0.01). The interpretation of the differences between the patterns was further described by effect sizes (Cohen’s *d*: ≥ 0.20 small effect, ≥ 0.50 medium effect, ≥ 0.80 large effect; Cohen, 1988).

To model the relationship between profile memberships and the index of lifelong LTPA controlling for age, auxiliary conditional effects models (similar to an ANCOVA, McLarnon and O’Neill, 2018) were applied. Controlling for age was important as age-related differences in the life course can occur, which is already partly considered in the index of lifelong LTPA. To interpret the mean differences between profiles, again the Wald test indicated significance (*p*-value < 0.01), and the Cohen’s *d* indicated effect size. All analyses were performed with Mplus (Muthén and Muthén 1998–2017) using the maximum likelihood estimation with robust standard errors.

## Results

### Sample characteristics

The mean age at the time of the survey of the *n* = 1519 participants was 59.2 ± 11.75 years and 947 women (62.3%) were in the sample. Regarding the educational level, the mean of the 5‑level scale (1–5) was exactly 3 ± 1.22. Further general sample characteristics can be found in Supplementary Table 2. The means of the indicators and the index of lifelong LTPA are presented in Supplementary Table 3. Regarding the specific types of LTPAs, the first two columns in Supplementary Table 4 reflect the frequencies in the ten categories.

### The optimal profile solution

**Fig. 1:**
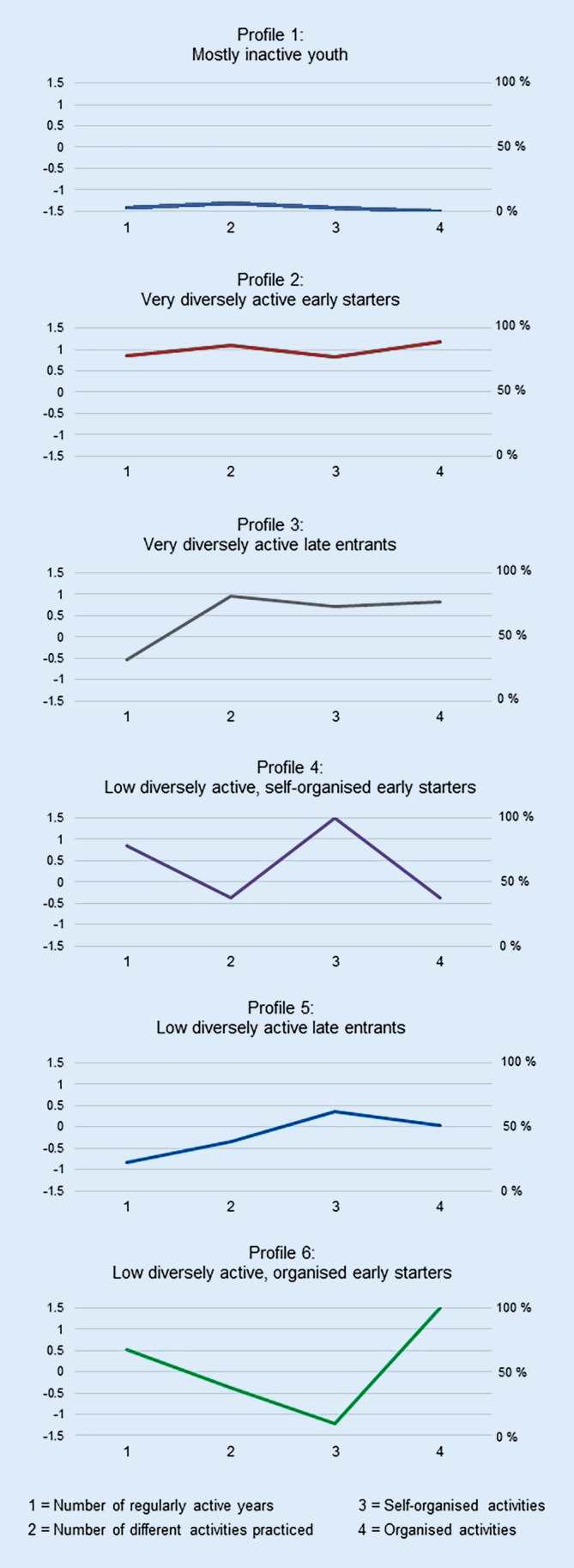
Plots of the six profiles. The left-sided y‑axis displays *z*-scores of the first two indicators (number of regularly active years and number of different activities practiced), whereas the right-sided y‑axis shows the percentages of people who practiced LTPA in this organisational setting for the third (self-organised activities) and fourth (organised activities) indicators

In a first step, the optimal number of profiles had to be determined. Therefore, the fit-indices for one to eight profiles are shown in Supplementary Table 5. In Supplementary Fig. 1, the fit-indices are plotted and the best-fitting solution is provided by the profile after which the slope flattens out. Therefore, five to seven profiles seemed to fit best. Considering content-related criteria, in terms of parsimony the five-profile solution would have been favoured. But regarding the qualitative differences within profile solutions, the six-profile solution showed more distinct patterns (Fig. 1) in comparison to the five and seven profile solutions (not shown). Furthermore, rather in the six-profile solution than in the five-profile solution theoretically plausible profiles emerged, and no profiles with less than 5% of the sample occurred. Altogether, the six-profile solution was seen as the best-fitting solution considering all criteria mentioned. This solution also showed a high value of the classification precision (Entropy = 0.86; see Supplementary Table 5).

### Description of the profiles

Based on RQ 1, the six profiles are plotted in Fig. 1. Table 1 shows per profile values of the indicators and sociodemographic information, and the categories of types of LTPAs are presented in Supplementary Table 4 (RQ 2).

**Table 1:** Description of the six profiles with indicators for the latent profile analysis (LPA) and further descriptive values (*n* = 1519)

Variables	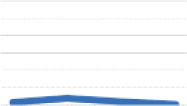 Profile 1	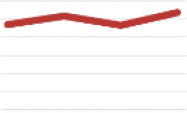 Profile 2	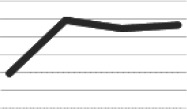 Profile 3	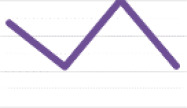 Profile 4	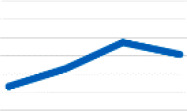 Profile 5	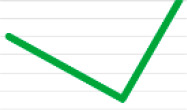 Profile 6	Entire sample
*n* (%)	293 (19.3%)	473 (31.1%)	117 (7.7%)	187 (12.3%)	220 (14.5%)	229 (15.1%)	1519 (100%)
*Four indicators for the LPA*							
Number of regularly active years (0–18)	0.05	13.53	5.29	13.62	3.46	11.54	8.5
Number of different activities practiced (0–5)	0.32	4.41	4.19	1.92	1.94	1.93	2.55
Self-organised activities^a^ (%)	2.9	77.6	73.9	> 99	62.3	0.9	53
Organised activities^a^ (%)	0.2	89.4	77.6	37.9	51.1	> 99	61
*Sociodemographic values*^b^							
*n* female (%)	226 (77.1)	275 (58.1)	60 (51.3)	142 (75.9)	120 (54.5)	124 (54.1)	947 (62.3)
Age (at time of the survey)	63.37	55.78	59.86	59.59	60.84	58.88	59.2
Educational level (1–5)	2.54	3.27	3.11	2.9	2.93	3.16	3.0

Means per profile are provided, the last column relates to the means of the whole sample to compare the values better directly from the profiles. Additional descriptive information for the whole sample can be found in Supplementary Table 1 and Supplementary Table 3

^a^The percentages mean the proportion of people practicing leisure-time physical activity (LTPA) in the given organisational setting compared to not practicing LTPA in this setting. This is equivalent to the probability of practicing LTPA in this organisational setting

^b^Statistical differences of the sociodemographic values between the respective profiles are shown in Supplementary Tables 6–8

In the following paragraphs, each profile is described by indicators (RQ 1) and further variables (RQ 2). Regarding the sociodemographic information as further variables, the significant differences and effect sizes between the profiles are shown in Supplementary Tables 6–8. Overall, despite a lot of significant differences between the profiles which might be caused by the large sample size, the effect sizes were negligible to small. Only one difference per sociodemographic variable revealed a moderate effect size (Supplementary Tables 6–8).

Profile 1 is called *mostly inactive youth* and includes participants who were inactive or very low active in youth. Compared to the other profiles, more women are in this profile, they are on average older, and lower educated. The *very diversely active early starters *reflect profile 2. This largest profile contains high and above-average values for all indicators. Thus, people of this profile started early in life with regular LTPAs and practiced several different activities in organised as well as self-organised settings. They are on average younger, higher educated and practiced each category of specific types of LTPAs above-average, especially walking and endurance activities, outdoor- and mountain activities and sports games. Profile 3 includes the *very diversely active late entrants*. This profile is quite similar to profile 2 with one difference: People in profile 3 did not have that many regularly active years during youth and are therefore called late entrants. Furthermore, in comparison to the other profiles, they are very often men and showed a very similar distribution of specific types LTPAs practiced as profile 2. The *low diversely active, self-organised early starters *in profile 4 began very early with regular LTPA but did not practice many different LTPAs in a self-organised and in a partially organised setting. Compared to the other profiles, many women are in this profile, and they were predominantly active in walking and endurance activities as well as outdoor- and mountain activities, but not frequently in sports games. Profile 5 represents the *low diversely active late entrants*. This profile is characterised by few regularly active years in few different LTPAs (primarily walking and endurance activities and outdoor- and mountain activities) partially practiced in self-organised and organised settings. Profile 6 is the *low diversely active, organised early starters. *People in this profile practiced few different LTPAs in an organised setting and entered LTPA rather early in youth. They practiced mainly sports games and comparatively less walking and endurance activities or outdoor- and mountain activities.

### Association between the profiles and lifelong LTPA

**Fig. 2:**
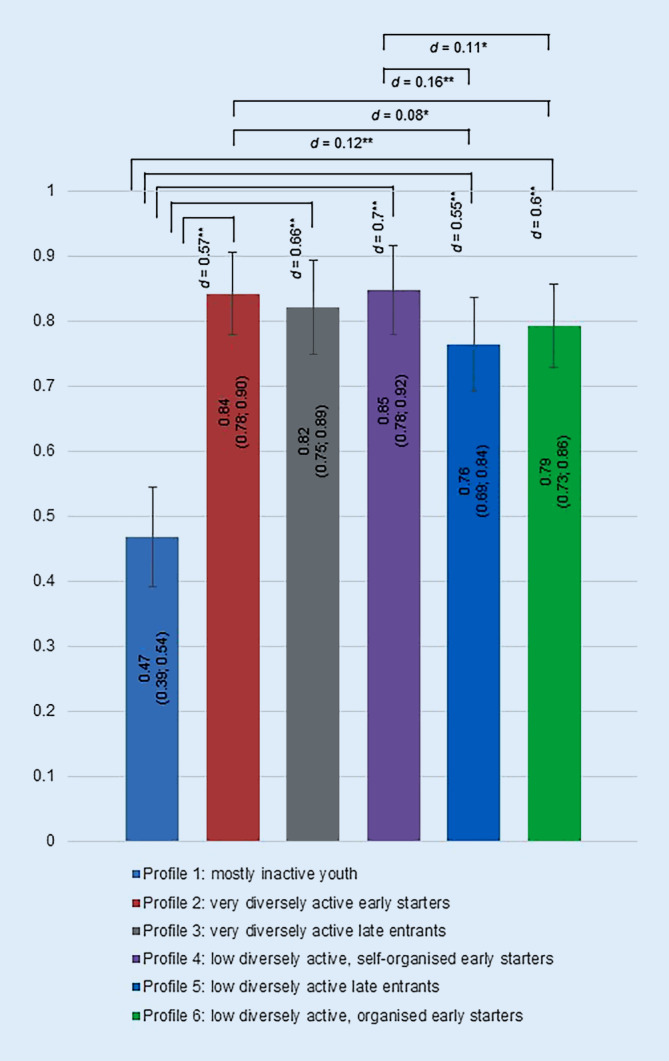
Means and 95% confidence intervals of the *Index of the lifelong LTPA* per profile, controlled for age. Comparisons between profiles contain the significance levels *p < 0.01, **p < 0.001 and effect sizes of Cohen’s *d*

Regarding RQ 3, Fig. 2 reveals the *Index of lifelong LTPA *per profile including significant differences and effect sizes, controlled for age. The most active people in adulthood were the *low diversely active, self-organised early starters *(profile 4, index = 0.85) and the *very diversely active early starters *(profile 2, index = 0.84). They were significantly more active than people from profile 1 with a medium effect size (for profile 4: *p* < 0.001, *d* = 0.7; for profile 2: *p* < 0.001, *d* = 0.57) and also significantly more active with a very small effect size than people from profile 5 (for profile 4: *p* < 0.001, *d* = 0.16; for profile 2: *p* < 0.001, *d* = 0.12) and profile 6 (for profile 4: *p* < 0.01, *d* = 0.11; for profile 2: *p* < 0.01, *d* = 0.08). Also mostly active in adulthood were the *very diversely active late entrants* (profile 3, index = 0.82), the *low diversely active, organised early starters *(profile 6, index = 0.79) and the *low diversely active late entrants *(profile 5, index = 0.76). They were all significantly more active with a medium effect size compared to people from profile 1 (for profile 3: *p* < 0.001, *d* = 0.66; for profile 6: *p* < 0.001, *d* = 0.6; for profile 5, *p* < 0.001, *d* = 0.55). The *mostly inactive youth *(profile 1) had the lowest activity ratio in adulthood (index = 0.47).

## Discussion

The present study investigated the LTPA behaviour in relation to time- and context-related aspects in youth and the association to lifelong LTPA in adulthood. Using retrospective life course data with a validated questionnaire and a person-oriented approach, qualitatively distinct patterns of LTPA in youth emerged. These patterns are related to different levels of lifelong LTPA in adulthood.

### Patterns of LTPA in youth

Time- and context-related aspects of LTPA in youth were applied to build patterns of LTPA behaviours. In the person-oriented approach used, the relevant variables can interact within individuals and can differ between individuals (Bergman et al., 2003), describing the heterogeneity of LTPA behaviours occurring in youth by identifying homogeneous subgroups (e.g. Gut et al., 2020). Based on the profiles, similarities to other studies can only be identified to a certain extent as the indicators used have never been studied in this context. Nevertheless, the *mostly inactive youth* (profile 1) emerged in other studies as well (Agans et al., 2017; Gut et al., 2020, 2022; Lawler et al., 2017). A simultaneously high level of breadth and depth of LTPA, similar to individuals in profile 2, has already been identified by Agans et al. (2017). Furthermore, doing LTPA in self-organised and organised settings (people in profiles 2 and 3) was already shown by Lawler et al. (2017), although profiles 2 and 3 differ regarding the depth of activities. Practicing LTPA in a predominantly self-organised setting (profile 4) has also been shown by other studies (Gut et al., 2020, 2022; Lawler et al., 2017), and conversely, doing LTPA in a predominantly organised setting (profile 6) has similarly been found by other research (Agans and Geldhof, 2012; Borgers et al., 2015; Gut et al., 2020, 2022; Lawler et al., 2017). Our data indicate that focusing on one organisational setting (profiles 4 & 6) was associated with practicing few different LTPAs. Consequently, following the already introduced understanding of human development (Lerner, 2006), the context of behaviour plays an important role for development in youth activities too (Zarrett et al., 2009). The patterns found in this study revealed that practicing LTPA can occur in a variety of contexts—between and within individuals. This is reflected in our patterns by differing the organisational setting and the breadth of activities as well as the depth of activities.

### Relationship between LTPA in youth and lifelong LTPA

Regarding the relationships with lifelong LTPA, the assumption of dependencies over the life course (Bernardi et al., 2019) can be supported by our data. Furthermore, in line with the understanding of human development (e.g. Lerner, 2006), there is not one activity according to LTPA in youth for conducive development (cf. Coakley, 2011) but interindividual differences in LTPA behaviours in youth. Thus, there are different ways to achieve a high level of lifelong LTPA, such as having high values of all indicators (profile 2) or high levels of at least two indicators (profiles 3, 4, & 6). If the *number of regularly active years* and the* number of different activities practiced* was rather low in youth, it is more difficult to achieve many active years in adulthood (profile 5). And mostly inactive people in youth were clearly the least active in adulthood (profile 1). Regarding the well-known social inequalities in practicing LTPA and sports (e.g. Rohrer & Haller, 2015; Scheerder, Vanreusel, Taks, & Renson, 2002), profile 1 displayed a comparatively low educational level and high proportion of women, whereas profile 5 with the second-lowest index of lifelong LTPA did not reflect these social differences. In addition, for example, individuals in profile 4 with the highest activity index over the life course had the second-lowest value regarding educational level and the second-highest proportion of women. Consequently, social inequalities cannot systematically explain the relationship between the LTPA behaviour in youth and lifelong activity.

Considering the differently shaped profiles in youth, it appears that certain indicators can compensate for each other. More specifically, it is evident that a high level of either *number of regularly active years *(> 13) or *number of different activities practiced *(> 4) in youth is crucial to becoming physically active in 80% of the years lived in adulthood (*Index of lifelong LTPA* > 0.80, see profiles 2, 3, 4). Thus, a high value of one of these indicators seems important, but they can compensate for each other. The importance of these two indicators is supported by variable-oriented studies (e.g., correlation or regression analyses) investigating them separately as predictors for lifelong LTPA (Batista et al., 2019; Engström, 2008; Cleland et al., 2012; Kjønniksen et al., 2008), which leads to the conclusion that a high value for both variables in youth is beneficial for lifelong LTPA. Our data also showed that a high value in both variables is beneficial (profile 2), indicating a synergistic effect; however, a compensation mechanism (profiles 3 and 4, and to some extent profile 6) is possible as well. From a theoretical understanding, the importance of the breadth of activities in youth is in line with the early sampling approach (Côté et al., 2007) and the *ability and readiness hypothesis *(Telama, 2009). The relevance of an early start or many physically active years, respectively, in youth is accompanied by time-related dependencies in the life course approach, more precisely the amount of time spent in certain situations or states influences the further course of life (Mayer, 1990), and in the meaning of an early socialisation, early-onset experiences in youth are relevant for shaping lifelong behaviour (Kirk, 2005; Sawyer et al., 2012). Furthermore, regarding organisational settings, being physically active in both settings (organised and self-organised) seems promising regarding lifelong LTPA (see profile 2 and 3), whereas practicing it in a predominantly self-organised setting goes hand-in-hand with a high activity index in adulthood as well (profile 4). However, being physically active only in an organised setting in youth reduced the likelihood to become comparatively very active during adulthood (see profile 6). A possible explanation could be that in adulthood, a more self-organised setting is preferred (Eime et al., 2015, 2020), and thus the organisational change from an only organised setting in youth to a rather self-organised setting in adulthood is accompanied by less activity. The most frequently practiced activities in profile 6 are sports games, predominantly practiced in a club, and maybe such activities are more difficult to continue in a rather self-organised setting in adulthood (cf. Downward, Lera-Lopez, & Rasciute, 2014). This could be an explanation for why the *low diversely active, self-organised early starters *(profile 4) are slightly more active during adulthood. In addition, the *carry-over value hypothesis *(Telama, 2009) may explain the relationship between practicing in a self-organised context in youth and the high level of LTPA during adulthood insofar as so-called lifetime activities (e.g. skiing, swimming or cycling; see categories walking and endurance activities and outdoor- and mountain activities in Supplementary Table 4) were practiced in this setting and can easily be continued during adulthood (see profile 4, but also profiles 2 and 3).

Overall, it should be noted that the differences regarding LTPA during adulthood between profiles 2 to 6 were low. However, when looking at the differences between the profiles 2 to 6 and profile 1, the differences were higher with medium effect sizes. Consequently, to stay active in adulthood any kind of regular LTPA in youth is better, respectively linked to a higher probability, than none. Yet, some profiles are particularly promising in order to reach a mainly continuous LTPA throughout adulthood.

### Limitations and future research

Some limitations must be considered for this study. LTPA was measured by self-report and retrospectively. Considering this method is not the most valid with a tendency to overestimate physical activity (Ainsworth & Levy, 2004), efforts were made to gather the most reliable and valid data possible (e.g. reliability test, see Lenze et al., 2021 for further information). Likewise, the frequency and intensity of LTPA were not captured due to the inaccuracy of measuring them in retrospective studies (Ropponen, Levälahti, Simonen, Videman, & Battié, 2001); rather, the regular practice of LTPA in terms of years was captured. Related to this, we are aware that older adults are more prone to a possible recall bias. However, our reliability test including older adults revealed good reliability values (see Supplementary Table 1). In addition, the activity level of the sample regarding LTPA was slightly higher than for the population in Switzerland (see Lamprecht et al., 2020), but a broad range of the Swiss population was covered with the aim of describing associations in general while not drawing conclusions about the entire population. Moreover, the sample of this study contained a broad age range, meaning that not all participants have already experienced the same life stages. To counteract this, the age of the participants was controlled for the analysis with lifelong LTPA, but not for the profile analysis. However, associated to this, a time-historical effect for the broad age range in this sample cannot be ruled out. Further sociodemographic variables such as sex and educational level were not directly controlled for but showed no systematic effect regarding the relationship to lifelong LTPA, as previously mentioned. Furthermore, the indicators used for the profile analyses were carefully deduced theoretically and empirically and comprise a broad spectrum of LTPA behaviours in relation to time- and context-related aspects over the first 20 years of life from a mainly sociological perspective, but they reflect not detailed and highly differentiated LTPA behaviours, such as specific organised settings of LTPA (e.g. sport club, fitness centre) or psychological aspects of LTPA (e.g. motivation, see Schmid, Gut, Yanagida, & Conzelmann, 2020). Lastly, our data reflect the sport system and culture over several decades in Switzerland, and therefore caution is required for a generalisation to other countries.

These limitations must be considered for implications, and consequently, future research should examine the results found with prospective data, other aspects of LTPA behaviour and in other countries to determine whether similar patterns and relationships to LTPA over the life course would emerge and to better understand the interdependencies over the life course.

## Conclusion

In terms of LTPA, being mostly inactive in youth was associated with low lifelong activity, whereas various profiles of LTPA in youth were related to a high or very high activity index over the life course. Thus, there is not just one way for lifelong activity. Considering interacting time- and context-related factors within persons and interindividual differences, multiple constellations in youth were associated with a very high activity index throughout life: a high value for each indicator; either a high depth or breadth of activities, combined with multiple organisational settings or particularly with a self-organised, and somewhat less with an organised setting. Thus, the findings of this study prove beneficial for the promotion of LTPA in youth and consequently over the entire life course.

### Supplementary Information

The Supplementary Material contains information about the reliability of the questionnaire used (Supplementary Table 1), about the sample (Supplementary Table 2), about the indicators (Supplementary Table 3) and types of LTPAs (Supplementary Table 4) used for the analysis, about the statistical analysis (Supplementary Table 5 and Supplementary Figure 1), and about inferential statistical differences between the profiles including effect size for sex (Supplementary Table 6), age (Supplementary Table 7), and educational level (Supplementary Table 8).

## Declarations

All studies mentioned were in accordance with the ethical standards and a consent statement was provided in each case.
