# Supplementary material for: The role of leisure-time physical activity in youth for lifelong activity—a latent profile analysis with retrospective life course data
Source: Ger J Exerc Sport Res. 2023 May 26;54(2):192–200. doi: 10.1007/s12662-023-00884-9 (PMC11135187; doi:10.1007/s12662-023-00884-9)
Supplement: Supplementary file 1 — The Supplementary Material contains information about the reliability of the questionnaire used (Supplementary Table 1), about the sample (Supplementary Table 2), about the indicators (Supplementary Table 3) and types of LTPAs (Supplementary Table 4) used for the analysis, about the statistical analysis (Supplementary Table 5 and Supplementary Figure 1), and about inferential statistical differences between the profiles including effect size for sex (Supplementary Table 6), age (Supplementary Table 7), and educational level (Supplementary Table 8). [file 12662_2023_884_MOESM1_ESM.docx]

**Supplementary Material**

For the main article:

**The role of leisure-time physical activity in youth for lifelong activity – A latent profile analysis with retrospective life course data**

Lars Lenze^a,b^, Claudia Klostermann^b^, Julia Schmid^a^, Markus Lamprecht^c^, Siegfried Nagel^a^

^a^Institute of Sport Science, University of Bern, Bern CH, 3012, Switzerland

^b^University of Teacher Education, University of Applied Sciences and Arts Northwestern Switzerland, Windisch CH, 5210, Switzerland

^c^Lamprecht und Stamm Sozialforschung und Beratung, Zurich CH, 8032, Switzerland

*Corresponding author*

Lars Lenze

Bremgartenstrasse 145, CH-3012 Bern

email: lars.lenze@unibe.ch

ORCID: 0000-0001-7610-8913

**Supplementary Table 1:** Test-retest reliability values from n = 29 persons, the two questionnaires were conducted 3 months apart.

| Variable | Scale | Krippendorff’s alpha | |
| --- | --- | --- | --- |
|  |  | *Point estimate* | *Bootstrap 95%–CI^a^* |
| *Regularly active years over the life course^b^* |  |  |  |
| First time taking up LTPA in life course (age) | ratio | .81 | .72 – .89 |
| Interruption(s) of LTPA in life course (yes/no) | nominal | .90 | .70 – 1.00 |
| Number of interruptions of LTPA in life course | ratio | .88 | .67 – 1.00 |
| Timing of interruptions of LTPA in life course (age) | ratio | .98 | .94 – 1.00 |
| *LTPAs practiced in youth* |  |  |  |
| Taking up current LTPAs (specific activities; if entered in first 20 years of life)^c^ | ratio | .92 | .86 – .97 |
| Number of activities practiced in youth (and not currently practiced)^c^ | ratio | .91 | .84 – .96 |
| Specific type of LTPAs practiced in youth | nominal | .95 | .89 – .99 |
| *Organisational setting of LTPA in youth* |  |  |  |
| Organisational forms practiced in youth^d^ | nominal | .93 | .87 – .99 |

Note: Krippendorff’s alpha is for a nominal scale similar to Scott’s Pi and for an interval scale similar to Pearson et al.’s intraclass–correlation coefficient (see Hayes & Krippendorff, 2007 for further information).

^a^As suggested (Hayes & Krippendorff, 2007), 10’000 bootstrap sampling distributions were done (CI = confidence interval).

^b^From this block of questions, it was determined for each person for each year of life whether they were physically active or inactive. The variables Number of regularly active years and Index of lifelong LTPA were built out of these questions.

^c^From these two questions, the variable *Number of different activities practiced* was built.

^d^Different organisational settings could be selected (multiple selection possible), thus for calculate Krippendorff’s alpha they were taken together which led to the two variables *Self-organised activities* and *Organised activities*.

**Supplementary Table 2:** Sample characteristics (n = 1519).

| Variable | *n* (%) | Mean | *SD* |
| --- | --- | --- | --- |
| *Sociodemographic information* |  |  |  |
| Sex |  |  |  |
| Male | 572 (37.7) |  |  |
| Female | 947 (62.3) |  |  |
| Age at time of the survey (Range 25-76) |  | 59.20 | 11.75 |
| For more information about the age distribution: 5 categories |  |  |  |
| 25–34 | 63 (4.1) |  |  |
| 35–44 | 120 (7.9) |  |  |
| 45–54 | 278 (18.3) |  |  |
| 55–64 | 476 (31.3) |  |  |
| 65-76 | 582 (38.3) |  |  |
| Level of education (5 categories) |  | 3 | 1.22 |
| 1 compulsory school | 74 (4.9) |  |  |
| 2 Secondary school/ lower professional education | 646 (42.5) |  |  |
| 3 Higher professional education leaving certificate | 263 (17.3) |  |  |
| 4 Technical college | 274 (18) |  |  |
| 5 University | 262 (17.2) |  |  |
| *Additional information about the sample* |  |  |  |
| At the time of the survey regularly active (LTPA) | 1419 (93.4) |  |  |
| Over the whole life course inactive (LTPA) | 44 (2.9) |  |  |
| Until 20 years of age inactive (LTPA) | 235 (15.5) |  |  |
| From 21 years until the current age inactive (LTPA) | 52 (3.4) |  |  |

Note: SD = Standard Deviation.

**Supplementary Table 3:** Descriptive data for the four indicators for the latent profile analysis and the related outcome variable (n = 1519).

| Variables | Mean | *SD* | *Min* | *Max* |
| --- | --- | --- | --- | --- |
| *Indicators for the latent profile analysis* |  |  |  |  |
| Number of regularly active years | 8.50 | 5.95 | 0 | 18 |
| Number of different activities practiced | 2.55 | 1.71 | 0 | 5 |
| Self-organised activities^a^ | 0.53 | 0.50 | 0 | 1 |
| Organised activities^a^ | 0.61 | 0.49 | 0 | 1 |
| *Auxiliary respective outcome variable* |  |  |  |  |
| Index of lifelong LTPA | 0.86 | 0.26 | 0 | 1 |

Note: SD = Standard Deviation.

^a^These variables are dummy coded and only contain values of 0 or 1. Thus, the Mean can interpreted as probability to be active in this organisational form, as Mplus uses it.

**Supplementary Table 4:** Types of LTPAs for the total sample (*n* = 1519) and per profiles, separated by category. Percentage value indicates the ratio of people in the respective (sub-)group practiced these types of LTPA in youth.

| Types of LTPAs | Entire  sample | 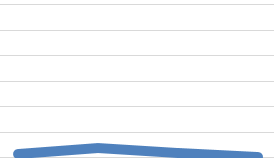  Profile 1 | 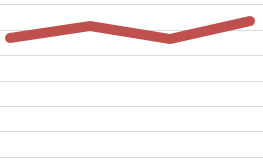  Profile 2 | 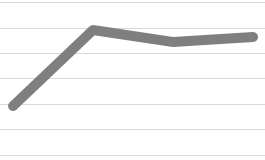  Profile 3 | 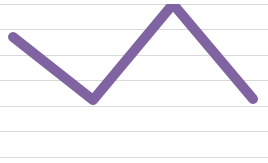  Profile 4 | 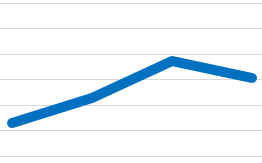  Profile 5 | 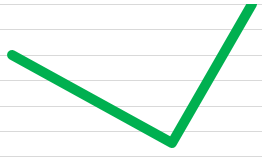  Profile 6 |
| --- | --- | --- | --- | --- | --- | --- | --- |
| Walking and endurance activities (e.g., running, swimming, cycling) | 60.1 % | 15.7 % | 88.6 % | 90.6 % | 65.2 % | 61.8 % | 36.7 % |
| Fitness (e.g., weight training) | 4.4 % | 0.7 % | 7 % | 10.3 % | 1.6 % | 5.5 % | 2.2 % |
| Gymnastics and multi-sport activities | 19.3 % | 4.1 % | 29.2 % | 25.6 % | 9.1 % | 14.1 % | 28.4 % |
| Athletics | 6.6 % | 0 % | 12.9 % | 12 % | 1.1 % | 3.2 % | 7.4 % |
| Compositional-creative activities (e.g., dancing) | 17 % | 2.7 % | 29.6 % | 29.1 % | 9.1 % | 9.5 % | 16.6 % |
| Release-oriented activities (e.g., yoga) | 0.7 % | 0 % | 1.3 % | 2.6 % | 0 % | 0.5 % | 0.4 % |
| Outdoor- and mountain activities (e.g., skiing) | 50 % | 11.9 % | 77 % | 82.9 % | 50.8 % | 43.6 % | 31.9 % |
| Sports games (e.g., football) | 32.1 % | 1.7 % | 49.7 % | 42.7 % | 18.2 % | 28.6 % | 44.1 % |
| Martial arts (e.g., taekwondo) | 6.1 % | 0 % | 9.9 % | 13.7 % | 2.7 % | 5.5 % | 5.7 % |
| Equestrian | 4.1 % | 0.7 % | 8.7 % | 3.4 % | 3.7 % | 1.4 % | 2.2 % |

Note: Profiles with a higher value in “number of different activities” (2^nd^ indicator) have thus higher percentage rates of activity categories in general (e.g., profile 2 & 3) than profiles with a lower value in the indicator mentioned (e.g., profile 1 or 6), considering that up to five activities per person can be indicated. *Walking and endurance activities* and *outdoor- und mountain activities* are practiced most often, which is not surprising for Switzerland (Lamprecht et al., 2020).

**Supplementary Table 5:** Fit-indices for the 1 to 8 profile of latent profile analyses (class-invariant, diagonal Σ; n = 1519).

| Model | LL | npar | AIC | CAIC | BIC | SABIC | BLRT | VLMRT | Entropy |
| --- | --- | --- | --- | --- | --- | --- | --- | --- | --- |
| 1 | -9898.819 | 6 | 19809.64 | 19847.59 | 19842 | 19822.53 |  |  | 1 |
| 2 | -9065.433 | 11 | 18152.87 | 18222.45 | 18211 | 18176.51 | < .001 | < .001 | 0.89 |
| 3 | -8787.108 | 16 | 17606.22 | 17707.43 | 17691 | 17640.6 | < .001 | < .001 | 0.902 |
| 4 | -8631.701 | 21 | 17305.4 | 17438.24 | 17417 | 17350.53 | < .001 | < .001 | 0.919 |
| 5 | -8537.834 | 26 | 17078.23 | 17292.14 | 17217 | 17134.11 | < .001 | < .001 | 0.928 |
| 6 | -8449.304 | 31 | 16960.61 | 17156.71 | 17126 | 17027.23 | < .001 | .001 | 0.862 |
| 7 | -8406.532 | 36 | 16809.14 | 17112.79 | 17001 | 16886.51 | < .001 | < .001 | 0.94 |
| 8* |  |  |  |  |  |  |  |  |  |

Note: LL: Log likelihood value; AIC: Aikake information criterion; CAIC: consistent AIC; BIC: Bayesian information criterion; SABIC: sample-sized adjusted BIC: BLRT: bootstrap likelihood ratio test: VLMRT: Vuong-Lo-Mendell-Rubin likelihood ratio test.

Significant BLRT and VLMR values mean a better-fitting solution than with one profile less. For the other fit-indices (LL, AIC, CAIC, BIC, SABIC), a lower value reflect a better fit, but with a large sample size, they tend to never reach a minimum and thus suggest always adding one profile (cf. Marsh et al., 2009). In this case, the fit-indices can be plotted to find the optimal solution by considering the elbow criterion (Wang & Morin, 2016). Additionally, the entropy value gives information on the precision of the classification of cases to profiles, whereby zero means a random classification and one a perfect classification (Masyn, 2013).

*convergence problems and thus not possible to calculate.**Supplementary Table 6:** Sex differences between the profiles indicated by Cohen’s d as the effect size and the Wald test for significant differences.

| Profiles  (means^a^) | 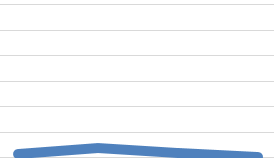  Profile 1 | 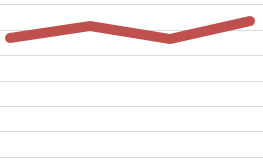  Profile 2 | 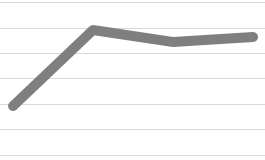  Profile 3 | 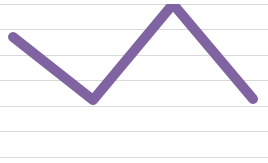  Profile 4 | 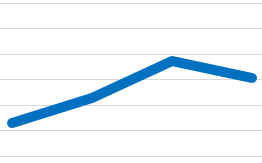  Profile 5 | 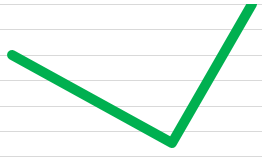  Profile 6 |
| --- | --- | --- | --- | --- | --- | --- |
| Profile 1  (0.775) | - | 0.363**  [0.216; 0.51] | 0.517**  [0.299; 0.734] | 0.034  [-0.15; 0.217] | 0.427**  [0.25; 0.604] | 0.478**  [0.303; 0.654] |
| Profile 2  (0.582) |  | - | 0.116  [-0.087; 0.318] | 0.309**  [0.139; 0.479] | 0.059  [-0.099; 0.217] | 0.066  [-0.092; 0.224] |
| Profile 3  (0.512) |  |  | - | 0.419*  [0.185; 0.652] | 0.052  [-0.171; 0.274] | 0.054  [-0.169; 0.277] |
| Profile 4  (0.759) |  |  |  | - | 0.354**  [0.159; 0.548] | 0.398**  [0.203; 0.593] |
| Profile 5  (0.546) |  |  |  |  | - | 0.003  [-0.18;0.186] |
| Profile 6  (0.544) |  |  |  |  |  | - |

Note: **p* < .01, ***p* < .001; 95% confidence interval of the effect size in brackets.

^a^Regarding sex, the mean indicates the proportion of women in the respective profile (coding: men = 0; women = 1).

**Supplementary Table 7:** Age differences between the profiles indicated by Cohen’s d as the effect size and the Wald test for significant differences.

| Profiles  (means^a^) | 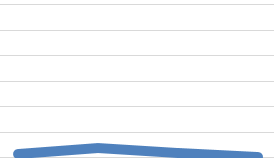  Profile 1 | 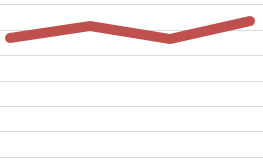  Profile 2 | 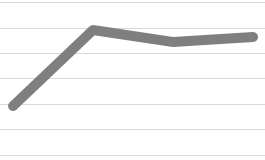  Profile 3 | 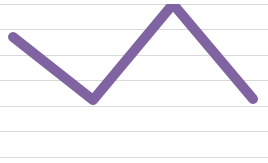  Profile 4 | 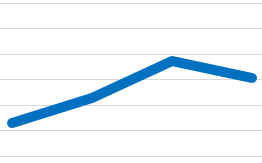  Profile 5 | 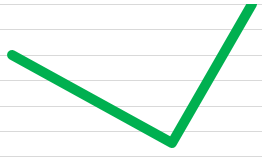  Profile 6 |
| --- | --- | --- | --- | --- | --- | --- |
| Profile 1  (63.37) | - | 0.616**  [0.467;0.765] | 0.328*  [0.113;0.544] | 0.346**  [0.161;0.53] | 0.224  [0.049; 0.399] | 0.386**  [0.211; 0.56] |
| Profile 2  (55.78) |  | - | 0.314*  [0.111; 0.517] | 0.298**  [0.128; 0.468] | 0.385**  [0.224; 0.547] | 0.242*  [0.084; 0.4] |
| Profile 3  (59.86) |  |  | - | 0.022  [-0.209; 0.253] | 0.076  [-0.301; 0.148] | 0.074  [-0.297; 0.148] |
| Profile 4  (59.59) |  |  |  | - | 0.098  [-0.293; 0.097] | 0.055  [-0.248; 0.139] |
| Profile 5  (60.84) |  |  |  |  | - | 0.148  [-0.333; 0.038] |
| Profile 6  (58.88) |  |  |  |  |  | - |

Note: **p* < .01, ***p* < .001; 95% confidence interval of the effect size in brackets.

^a^Regarding age, the mean reflects the mean age of the respective profile at the time of the survey.

**Supplementary Table 8:** Differences in educational level between the profiles indicated by Cohen’s d as the effect size and the Wald test for significant differences.

| Profiles  (means^a^) | 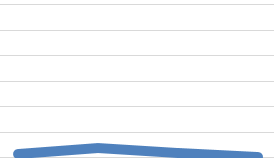  Profile 1 | 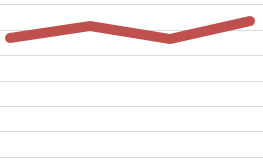  Profile 2 | 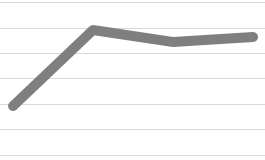  Profile 3 | 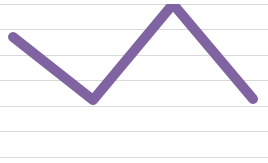  Profile 4 | 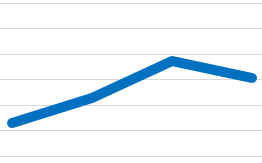  Profile 5 | 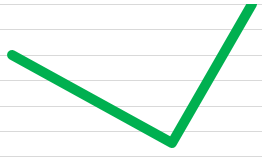  Profile 6 |
| --- | --- | --- | --- | --- | --- | --- |
| Profile 1  (2.54) | - | 0.554**  [0.406; 0.703] | 0.458**  [0.241; 0.674] | 0.29*  [0.096; 0.465] | 0.299*  [0.123; 0.476] | 0.475**  [0.3; 0.65] |
| Profile 2  (3.27) |  | - | 0.11  [-0.093; 0.312] | 0.27*  [0.1; 0.44] | 0.243*  [0.082; 0.403] | 0.078  [-0.08; 0.236] |
| Profile 3  (3.11) |  |  | - | 0.163  [-0.68; 0.394] | 0.135  [-0.09; 0.359] | 0.03  [-0.192; 0.253] |
| Profile 4  (2.9) |  |  |  | - | 0.24  [-0.219; 0.171] | 0.19  [-0.004; 0.383] |
| Profile 5  (2.93) |  |  |  |  | - | 0.162  [-0.23; 0.348] |
| Profile 6  (3.16) |  |  |  |  |  | - |

Note: **p* < .01, ***p* < .001; 95% confidence interval of the effect size in brackets.

^a^Regarding age, the mean reflects the mean educational level of the respective profile at the time of the survey.

**Supplementary Figure 1:** Plotted fit-indices for 1 to 7 profiles.

Note: As suggested from Morin & Wang (2016), these four fit-indices are plotted and the best-fitting solution is provided by the profile after which the slope flattens out (“elbow criterion”).

AIC: Aikake information criterion; CAIC: consistent AIC; BIC: Bayesian information criterion; SABIC: sample-sized adjusted BIC.
